# Function and molecular regulation of DWARF1 as a C-24 reductase in brassinosteroid biosynthesis in Arabidopsis

**DOI:** 10.1093/jxb/ery038

**Published:** 2018-02-08

**Authors:** Ji Hyun Youn, Tae-Woo Kim, Se-Hwan Joo, Seung-Hyun Son, Jeehee Roh, Sunyoung Kim, Tae-Wuk Kim, Seong-Ki Kim

**Affiliations:** 1 Department of Life Science, Chung-Ang University, Seoul 156-756, Republic of Korea; 2 Department of Life Science, Hanyang University, Seoul 133-791, Republic of Korea

**Keywords:** *Arabidopsis thaliana*, biosynthetic connections, brassinosteroids, BR C-24 reductase, DWARF1

## Abstract

DWARF1 (DWF1) is a sterol C-24 reductase that catalyses the conversion of 24-methylenecholesterol (24-MCHR) to campesterol (CR) in Arabidopsis. A loss-of-function mutant, *dwf1*, showed similar phenotypic abnormalities to brassinosteroid (BR)-deficient mutants. These abnormalities were reversed in the wild-type phenotype by exogenous application of castasterone (CS) and brassinolide (BL), but not dolichosterone (DS). Accumulation of DS and decreased CS were found in quantitative analysis of endogenous BRs in *dwf1.* The enzyme solution prepared from *dwf1* was unable to convert 6-deoxoDS to 6-deoxoCS and DS to CS, as seen in either wild-type or *35S:DWF1* transgenic plants. This suggests that DWF1 has enzyme activity not only for a sterol C-24 reductase, but also for a BR C-24 reductase that catalyses C-24 reduction of 6-deoxoDS to 6-deoxoCS and of DS to CS in Arabidopsis. Overexpression of *DWF1* in a BR-deficient mutant (*det2 35S:DWF1*) clearly rescued abnormalities found in *det2*, indicating that DWF1 functions in biosynthesis of active BRs in Arabidopsis. Expression of *DWF1* is down-regulated by application of CS and BL and in a BR-dominant mutant, *bes1-D*. E-boxes in the putative promoter region of *DWF1* directly bind to a BR transcription factor, BES1, implying that *DWF1* expression is feedback-regulated by BR signaling via BES1. Overall, biosynthesis of 24-methylene BR is an alternative route for generating CS, which is mediated and regulated by DWF1 in Arabidopsis.

## Teaser

DWARF1 functions as a brassinosteroid C-24 reductase that converts 24-methylene brassinosteroids to 24-methyl brassinosteroids to regulate the endogenous level of an active brassinosteroid, castasterone, to control growth and development in Arabidopsis.

## Introduction

**Fig. 1.**
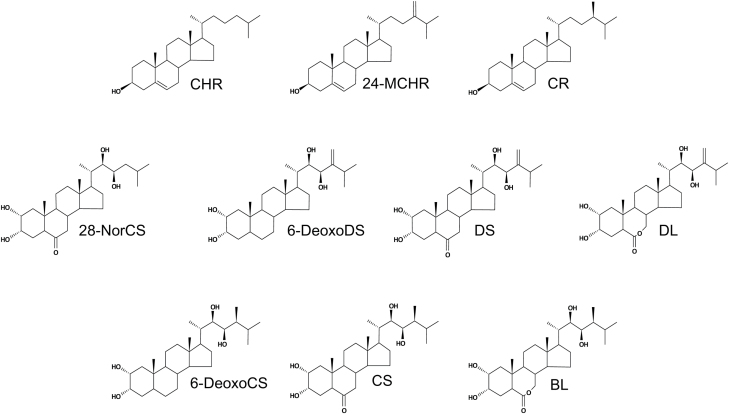
Structure of frequently mentioned phytosterols and BRs in the study. BL, brassinolide; CHR, cholesterol; CS, castasterone; CR, campesterol; 6-DeoxoCS, 6-deoxocastasterone; 6-DeoxoDS, 6-deoxodolichosterone; DL, dolicholide; DS, dolichosterone; 24-MCHR, 24-methylenecholesterol; 28-NorCS, 28-norcastasterone.

Plant growth and development are controlled by chemical signals synthesized in plants (Santner *et al.*, 2009; Wolters and Jürgens, 2009). Brassinosteroids (BRs) are endogenous steroid signals that regulate diverse phenomena at very low concentrations (>10^−8^ M) in processes related to the growth and development of plants, such as root development, stem elongation, leaf development, photomorphogenesis, vascular and floral development, and senescence (Clouse and Sasse, 1998; Fujioka and Yokota, 2003; Choe, 2004; Wu *et al.*, 2008; Kim and Wang, 2010). In addition, BRs are important factors in stress modulation and defense in plants (Krishna, 2003; Bari and Jones, 2009). Since brassinolide (BL) (Fig. 1) and castasterone (CS) were identified from pollen of *Brassica napus* and insect galls of *Castanea sativa*, over 50 plant steroids structurally related to them have been characterized from many plant tissues, ranging from lower to higher plants (Bishop and Yokota, 2001; Bajguz and Tretyn, 2003; Fujioka and Yokota, 2003). Therefore, BRs are regarded as essential plant hormones for normal growth and development.

Naturally occurring BRs can be classified into C_27_-, C_28_-, and C_29_-BRs based on the presence of alkyl groups occupying the C-24 position in 5α-cholestane carbon skeleton side chain (Yokota, 1997; Bajguz and Tretyn, 2003). C_27_-BRs carry no alkyl group at C-24 (28-norBRs). C_28_-BRs contain 24-methylene, 24*S*- and 24*R*-methyl BRs. C_29_-BRs include 24-ethylidene, 24-ethyl and 24-methylene-25-methyl BRs. The carbon skeletons of C_27_-, C_28_-, and C_29_-BRs are similar to those of 4-demethylsterols, which are abundant in plants. This implies that BRs are biosynthesized from 4-demethylsterols in plants (Fujioka and Yokota, 2003). Based on this speculation, multiple biosynthetic pathways that generate diverse kinds of BRs are operant in plants (Fujioka *et al.*, 1997; Yokota, 1997; Joo *et al.*, 2009).

To date, CS and BL have been the BRs most frequently identified from plants (Fujioka, 1999; Bajguz and Tretyn, 2003). Given their stronger biological activity and wider distribution in the plant kingdom than any other types of BRs, CS and BL are also considered the most important bioactive BRs in plants (Bajguz and Tretyn, 2003). CS and BL possess 24*S*-oriented methyl groups at C-24 positions, suggesting that they can be biosynthesized from campesterol (CR). Feeding experiments using CR and downstream intermediates in *Catharanthus roseus* indicated the presence of two parallel biosynthetic pathways for CS and BL generation, the early and late C-6 oxidation pathways (Suzuki *et al.*, 1993). Early and/or late C-6 oxidation have also been demonstrated in other higher plants, such as Arabidopsis, tomato, rice, maize, and *Phaseolus vulgaris*, as well as in lower plants such as *Marchantia polymorpha* (Kim *et al.*, 2001; Fujioka and Yokota, 2003; Kim *et al.*, 2004a, b, c; Kim *et al.*, 2005*c*). This suggests that early and late C-6 oxidation pathways are common biosynthetic routes for BRs in the plant kingdom. However, some evidence has accumulated that is contrary to the notion that early C-6 oxidation plays a role in CS and BL biosynthesis (Bishop *et al.*, 1999; Kim *et al.*, 2000; Noguchi *et al.*, 2000). The first reaction in the pathway is C-6 oxidation of campestanol (CN) to 6-oxoCN, which was established in *Catharanthus* crown gall cells (Fujioka *et al.*, 2000). Since that study, C-6 oxidation of CN to 6-oxoCN has not been confirmed in other plants. C-22 hydroxylation of 6-oxoCN to cathasterone (CT) has not been confirmed in *Catharanthus* crown gall cells, even though CT is endogenous to such cells (Fujioka *et al.*, 1995). Neither the conversion of 6-oxoCN to CT nor the presence of CT has been demonstrated in other plants (Joo *et al.*, 2002). Additionally, CYP90B1 (DWF4) hydroxylates CN to 6-deoxoCT, but does not convert 6-oxoCN to CT (Fujita *et al.*, 2006). These results indicate that the early C-6 oxidation pathway is commonly interrupted in plant tissues.

BRs are highly oxidized steroids. It is thus thought that the incorporation of oxygen atoms into phytosterols is catalysed by cytochrome P450 (CYP) monooxygenase in BR biosynthesis. Functional studies using heterogeneously expressed Arabidopsis CYPs in *Escherichia coli*, yeast, and insect cells have revealed that CYP90B1 (DWF4) catalyses C-22 hydroxylation of CR to 22-hydroxyCR (Supplementary Fig. S1A at *JXB* online), rather than catalysing C-22 hydroxylation of 6-oxoCN to CT or CN to 6-deoxoCT in the early and late C-6 oxidation pathways (Fujita *et al.*, 2006). CYP90A1 (CPD) mediates C-3 oxidation of 3β-hydroxylated intermediates to their corresponding 3-dehydro derivatives such as 22-hydroxyCR to 22-hydroxy-campesta-4-en-3-one, 6-deoxoCT to 22-hydroxy-campesta-3-one and 6-deoxo-teasterone (6-deoxoTE) to 6-deoxo-3-dehydroteasterone (6-deoxo3DT) (Ohnishi *et al.*, 2012). CYP90C1 and CYP90D1 have similar enzyme activities for C-23 hydroxylation in 6-deoxoCT to 6-deoxoTE, 22-hydroxy-campesta-3-one to 3-dehydro-6-deoxoTE, and 3-*epi*-6-deoxoCT to 6-deoxo-typhasterol (6-deoxoTY) (Kim *et al.*, 2005*a*, Ohnishi *et al.*, 2006). Although CYP85A2 has stronger enzyme activity than CYP85A1, both molecules catalyse C-6 oxidation of 6-deoxoTE, 3-dehydro-6-deoxoTE, 6-deoxoTY, and 6-deoxoCS to teasterone (TE), 3-dehydroTE, typhasterol (TY), and CS, respectively (Kim *et al.*, 2005*b*). Finally, CYP85A2 has been shown to convert CS to BL, which suggests that CYP85A2 is a bifunctional enzyme, not only a BR C-6 oxidase but also a BL synthase, in Arabidopsis plants (Kim *et al.*, 2005*b*).

Together with the presence of a biosynthetic sequence, CS and BL are biosynthesized by a new pathway called the CN-independent or early CR C-22 hydroxylation pathway (Supplementary Fig. S1A) in Arabidopsis (Fujita *et al.*, 2006). The pathway conversion diagram is as follows: CR→22-hydroxy-CR→22-hydroxy-campesta-4-en-3-one→22-hydroxy-campesta-3-one→6-deoxoCT and 3-*epi*-dexoxoCT. In the CN-independent pathway, conversion of 22-hydroxy-campesta-4-en-3-one to 22-hydroxy-campesta-3-one is catalysed by DET2, originally identified as a 5α-reductase for CR to CN conversion in plants (Fujita *et al.*, 2006).

DS is a C_28_-BR that carries an exo-methylene group at a C-24 side chain (Baba *et al.*, 1983). To date, DS and its biosynthetically related BRs, such as 6-deoxoDS and dolicholide (DL), have been identified from four dicots (Arabidopsis, *Phaseolus vulgaris*, *Dolichos purpreus* and *Vicia faba*), two monocots (rice and maize) and a pteridophyte (*Equisetum arrense*), suggesting that these 24-methylene BR molecules are common in the plant kingdom (Bajguz and Tretyn, 2003). In all plants from which DS has been identified, CS has also been identified. This suggests that the biosynthetic pathways that synthesize the two compounds might operate simultaneously in plants (Bajguz and Tretyn, 2003; Hwang *et al.*, 2009; Lee *et al.*, 2010). Recently, a crude enzyme solution prepared from Arabidopsis successfully mediated conversion of 6-deoxoDS to CS, intermediated by DS (Hwang *et al.*, 2009; Lee *et al.*, 2010). This suggests successful biosynthesis of 24-methylene BRs to 24-methyl BRs in plants. However, the enzyme responsible for C-24 reduction of 24-methylene BRs to 24-methyl BRs, a BR C-24 reductase, has not yet been identified in plants. This prompted us to identify a BR C-24 reductase in Arabidopsis. Here, biological and biochemical evidence for DWF1 as a BR C-24 reductase in Arabidopsis is presented. In addition, molecular regulation of *DWF1* expression in the plant is described.

## Materials and methods

### Plant materials and growth conditions

Seeds of Col-0, En-2, *dwf1*, *det2*, *35S:DWF1*, *det2 35S:DWF1*, *bes1-D*, and *bzr1-1D* were each surface-sterilized in ethanol–water (70:30, v/v), rinsed in distilled water (DW), cold-treated at 4 °C for 2 d and plated on 0.5× Murashige and Skoog (Duchefa, Haarlem, the Netherlands) medium containing 1% sucrose and 0.7% agar. Plates were kept in the light (120 μmol m^−2^ s^−1^) at 22 °C for 16 h and in the dark for 8 h in a growth chamber (Sanyo, Osaka, Japan).

### Analysis of BRs and sterols in Arabidopsis

Arabidopsis plants (30 kg) grown for 6 weeks on soil were harvested and extracted three times with 3 liters of 90% methanol. Evaporated extracts were partitioned three times between water (3 liters) and chloroform (3 liters). The chloroform-soluble fractions were concentrated and partitioned three times between 80% methanol (3 liters) and *n*-hexane (3 liters). The concentrated 80% methanol extracts were repartitioned three times between ethyl acetate (3 liters) and phosphate buffer (pH 7.8, 3 liters). Ethyl acetate-soluble residues were subjected to silica gel chromatography. Columns were eluted with 150 ml of chloroform containing 1, 2, 3, 4, 5, 6, 7, 8, 9, 10, 20, 50, or 100% (v/v) methanol. The 3–7% (v/v) methanol fractions were combined, concentrated and subsequently purified using a Sep-Pak C_18_ cartridge column (Waters, Milford, MA, USA) eluted with 0, 50, and 100% methanol (20 ml each). The 100% methanol fraction obtained from the Sep-Pak column was dried, dissolved in a small amount of methanol and then subjected to reversed-phase HPLC (Senshu-Pak C_18_, 10 × 150 mm). The column was eluted at a flow rate of 2.5 ml min^−1^ using different percentages of acetonitrile (MeCN) in water for different lengths of time: 0–20 min, 45% MeCN; 20–40 min, 45–100% MeCN; or 40–70 min, 100% MeCN. Fractions were collected every minute. Under the same HPLC condition, synthetic 6-deoxoDS, DS, and DL was detected in fractions 34–37, 14–15 and 9–10, respectively. These fractions were analysed by capillary GC-MS after bismethanboronation. Endogenous amounts of 6-deoxoDS and DS were calculated by a calibration curve established with a molecular ion *(m*/*z* 496 and *m*/*z* 510 for 6-deoxoDS and DS, respectively) in GC–selective ion monitoring (SIM) analysis. The *n*-hexane-soluble fraction was extracted with methanol:chloroform (4:1, v/v). Extracts were then concentrated and solvent-partitioned between chloroform and water. D_7_-Cholesterol (D_7_-CHR), 0.5 μg, was added to the chloroform-soluble fraction as an internal standard. The fraction extracted with *n*-hexane after alkaline hydrolysis was purified on a Sep-Pak C_18_ cartridge column and subjected to GC-MS analysis after trimethylsilylation. Endogenous amounts of 24-MCHR and CR were measured based on relative ratios of 24-MCHR and CR against endogenous amounts of cholesterol (CHR) with D_7_-CHR as an internal standard.

### Enzyme assay

Three-week-old light-grown plants (20 g) were homogenized with 0.1 M sodium phosphate buffer (pH 7.4) containing 1 mM EDTA, 1 mM DTT, 0.1 mM phenylmethylsulfonyl fluoride (PMSF), 15 mM 2-mercaptoethanol, 15% glycerol, 250 mM sucrose, 40 mM ascorbate, and 1% insoluble polyvinyl-polypyrrolidone, and centrifuged for 10 min at 8000 *g* to remove cell debris. Resulting supernatants were re-centrifuged for 30 min at 20 000 *g*. Following the addition of cold acetone (final volume 40%), precipitates were suspended in 0.1 M sodium phosphate buffer (pH 7.4) containing 1.5 mM 2-mercaptoethanol and 30% glycerol in order to create a crude enzyme solution. The protein concentration of the enzyme solution was estimated with a Bradford assay (Bio-Rad, Hercules, CA, USA) using bovine serum albumin as a standard.

Enzyme assays for metabolism of 6-deoxoDS and DS were initiated by the addition of substrates (5 μg each) to the enzyme solution (4–5 mg protein ml^−1^) in the presence of NADPH. Following incubation at 37 °C for 30 min, metabolites of enzyme reactions were extracted with ethyl acetate (1.2 ml) and concentrated. Ethyl acetate-soluble fractions were loaded onto Sep-Pak C_18_ cartridge columns and washed with aqueous methanol (in 5 ml 50% methanol, 5 ml of 60% and 5 ml of 80% methanol). The 80% methanol-eluted fractions were concentrated, dissolved in 50 μl of methanol and then subjected to reversed-phase HPLC (Senshu-Pak C_18_, 10 × 150 mm). Fractions were then eluted at a flow rate of 2.5 ml min^−1^ with the following MeCN-water gradient: 0–20 min, 45% MeCN; 20–40 min, 45–100% MeCN; and 40–70 min, 100% MeCN. Fractions were collected every minute. The HPLC fractions corresponding to authentic 6-deoxoCS, DS and CS in the same HLPC conditions were collected and analysed via GC-MS or GC-SIM.

### GC-MS and GC-SIM analysis

GC-MS and GC-SIM analyses were carried out on a Hewlett-Packard 5973 mass spectrometer (Electron impact ionization, 70 eV) coupled to an Agilent 6890 gas chromatograph (Palo Alto, CA, USA) fitted with a fused silica capillary column (HP-5, 0.25 mm×30 m, 0.25 μm film, Agilent). The oven temperature was maintained at 175 °C for 2 min, raised to 280 °C at a rate of 40 °C min^−1^ and then maintained at 280 °C. Helium was used as the carrier gas at a flow rate of 1 ml min^−1^ and samples were introduced using the on-column injection mode. Methaneboronation was carried out by heating samples dissolved in pyridine-containing methaneboronic acid (2 mg ml^−1^) at 70 °C for 30 min.

### RNA isolation of qRT-PCR

Total RNAs were extracted using TRI reagent (Sigma-Aldrich, St Louis, MO, USA) in accordance with the manufacturer’s instructions. For RT-PCR, 2 μg of total RNA was reverse-transcribed by M-MLV RT (Promega, Madison, WI, USA); 2 μl of the RT product was employed as a PCR template. Quantitative RT-PCR (qRT-PCR) was performed using iQ SYBR Green SuperMix and the iCycler iQTM RT-PCR Detection System (Bio-Rad). PCR conditions consisted of denaturation at 95 °C for 3 min, followed by 40 cycles of denaturation at 95 °C for 15 s, annealing at 58 °C for 15 s and extension at 72 °C for 30 s. A dissociation curve was generated at the end of each cycle to verify amplification of a single product. mRNA levels were quantified using the 2^−ΔΔ*C*T^method (Livak and Schmittgen 2001). Expression level of the target gene was normalized relative to the expression of the housekeeping gene *UBIQUITIN 5* (*UBQ5*). Gene-specific primers are described in Supplementary Table S1.

### Selection of *dwf1*, *35S:DWF1*, and *det2 35S:DWF1*

An Arabidopsis *dwf1* knockout mutant (Salk006932) with a transfer (T)-DNA insertion in the *DWF1* gene’s first exon was obtained. T-DNA insertion was confirmed using T-DNA LB primer (5′-CTTTGACG TTGGAGTCCACGTTCTTTAATA-3′) and *DWF1* reverse primer (Supplementary Table S1). For overexpression, *DWF1* full-length cDNA was amplified by PCR and cloned into pGEM-T Easy vectors (Promega), followed by sub-cloning into a binary vector (pBI121) driven by a constitutive 35S promoter (*35S:DWF1*). Transgene constructs were confirmed by sequencing and subsequently transformed into wild-type (T_0_) and BR-deficient mutant, *det2*, by the floral-dip method (Clough and Bent, 1998). T_1_ seeds with cold pretreatment were screened on 0.5× MS medium containing 50 μg l^−1^ kanamycin, 1% sucrose, and 0.7% agar. Homozygous lines resistant to kanamycin were obtained at the T_3_ generation.

Expression level of *DWF1* in wild-type, *dwf1*, *35S:DWF1*, and *det2 35S:DWF1* was measured by semi-quantitative RT-PCR (semi-qRT-PCR) using *DWF1* forward and reverse primers (Supplementary Table S1). Cycling conditions were as follows: 5 min at 95 °C, 35 cycles of 15 s at 95 °C, 15 s at 59 °C, and 15 s at 72 °C.

### Rescue experiments

For rescue experiments for *dwf1* and *det2*, DS, CS and BL were sprayed onto 21-day-old *dwf1* and *det2* plants 3–5 times at 5-day intervals. In hormone stocks, 5 μM of BRs were dissolved in ethanol containing 1% DMSO and 0.5% Tween-20 in ethanol. For seedlings, *dwf1* and *det2* were planted and grown in the dark on 0.5× MS medium containing 10^–9^ M CS, DS and BL, 1% sucrose, and 0.7% agar. Hypocotyl length was measured after 7 d.

### Electrophoretic mobility shift assay

Maltose binding protein (MBP) and MBP–BES1 proteins were expressed and affinity-purified from *E. coli* (BL21-DE3) using amylose resins (New England Biolabs, Ipswitch, MA, USA). The *DWF1* promoter fragments (WT probes) and mutated probes (MT probes, CANNTG to AAAAAA) used for electrophoretic mobility shift assay (EMSA) were prepared using primers described in Supplementary Table S1. Double-stranded DNA probes, 100 ng, were labeled with [^32^P]dATP using T4 polynucleotide kinase using a 5′ end labeling system (‘hot probes’). Unlabeled double-stranded DNA probes were used as ‘cold probes’. Binding reactions were carried out in 2 μl of 5× binding buffer (50 mM Tris–HCl pH 7.5, 250 mM NaCl, 2.5 mM EDTA, 2.5 mM DTT, 5 mM MgCl_2_, and 20% glycerol), 4 μl of 3 μg MBP-BES1 proteins and 3 μl of DW with a 1 μl probe. After 30 min of incubation at room temperature, reactions were resolved using 4% native polyacrylamide gels with 0.5× TBE buffer (containing 5.39 g l^−1^ Tris, 2.75 g l^−1^ boric acid and 0.37 g l^−1^ EDTA, pH 8.0) and exposed to a phosphor imaging screen.

### Chromatin immunoprecipitation

Chromatin immunoprecipitation (ChIP) was performed on 10-day-old *35S:YFP* and *BES1-YFP* overexpression transgenic lines. Seedlings (1 g) were cross-linked with 50 ml of 1% formaldehyde in a vacuum for 5 min. A total of 2.5 ml of 2 M glycine was added to quench cross-linking. After rinsing seedlings with DW, tissues were ground with liquid nitrogen and resuspended in cold extraction buffer I (0.4 M sucrose, 10 mM Tris–HCl, pH 8, 10 mM MgCl_2_, 5 mM 2-mercaptoethanol, 0.1 mM PMSF, and 1× protease inhibitor (Roche Diagnostics, Indianapolis, IN, USA)). Filtrates filtered through Miracloth (Calbiochem, Darmstadt, Germany) were centrifuged at 1800 *g* for 10 min at 4 °C. The pellet was resuspended in 1ml of extraction buffer II (0.25 M sucrose, 10 mM Tris–HCl, pH 8, 10 mM MgCl_2_, 1% Triton X-100, 5 mM 2-mercaptoethanol, 1 mM PMSF, and 1× protease inhibitor) and centrifuged at 14 000 *g* for 10 min at 4 °C. The pellet was resuspended in 600 μl of extraction buffer III (1.7 M sucrose, 10 mM Tris–HCl, pH 8, 0.15% Triton X-100, 2 mM MgCl_2_, 5 mM 2-mercaptoethanol, 1 mM PMSF, and 1× protease inhibitor) and centrifuged at 14 000 *g* for 1 h at 4 °C. The crude chromatin pellet was resuspended in 250 μl of nuclear lysis buffer (50 mM Tris–HCl, pH 8.0, 10 mM EDTA, 200 mM NaCl, 0.5% Triton X-100, 1 mM PMSF and 1× protease inhibitor) and sonicated with a Branson sonifier (VWR) to achieve an average fragment size of ∼0.5–1.0 kb. The solution obtained was centrifuged at 14 000 *g* for 10 min at 4 °C and supernatant was transferred to a new tube. Chromatin solution was diluted with an equal volume of DW and 50 μl was taken out to use as input. After pre-clearing with protein A-sepharose beads (Sigma-Aldrich), 5 μl of with yellow fluorescent protein (YFP)-specific monoclonal antibody (Thermo Fisher Scientific, Waltham, MA, USA) was added to chromatin solution and incubated overnight with rotating at 4 °C. The immunocomplexes were extracted by incubating with 50 μl of protein A beads for 1 h at 4 °C. The extract was washed with 250 μl of wash buffer (1% SDS and 0.1 M NaHCO_3_) and then reverse cross-linked with a final concentration of 200 mM NaCl at 65 °C for overnight. Extracted DNA using a gel elution kit (MP) was eluted by 50 μl of TE and used for PCR analysis. ChIP-PCR was performed using primers specific for *DWF1* and *SAUR-AC1* promoter regions (Supplementary Table S1). Cycling conditions were as follows: 3 min at 95 °C, 35 cycles of 10 s at 95 °C, 10 s at 52 °C, and 10 s at 72 °C. The ChIP experiments were performed three independent times.

## Results

### Identification and metabolism of 6-deoxoDS in Arabidopsis

The presence of DS was recently demonstrated with a large quantity (30 kg) of Arabidopsis (Lee *et al.*, 2010). The presence of DS analogs, especially 6-deoxoDS and DL, in Arabidopsis extract was investigated in order to understand the biosynthesis of DS. Following reversed-phase HPLC, the fractions corresponding to synthetic 6-deoxoDS and DL were collected and analysed by capillary GC-MS/SIM after derivatization to bismethaneboronates (BMBs). BMBs, thought to be a form of 6-deoxoDS, exhibited characteristic molecular ions for 6-deoxoDS-BMB at *m*/*z* 496 (M^+^), 356, 342, 327, 273, 153, and 124 in HPLC fractions at GC retention times similar to authentic 6-deoxoDS-BMB, verifying that the compound is 6-deoxoDS (Table 1). However, BMBs in compounds of HPLC fractions equivalent to synthetic DL failed to show any characteristic molecular ions for DL-BMB, suggesting that DL was not present in Arabidopsis or that endogenous DL levels in Arabidopsis were too low to be detected by GC-MS/SIM. Endogenous levels of 6-deoxoDS and DS in Arabidopsis were measured by GC-SIM-based calibration curves using molecular ions. Arabidopsis contains approximately 0.13 and 0.01 ng g^−1^ fresh weight of 6-deoxoDS and DS, respectively (Table 1).

**Table 1.** HPLC and GC data for 6-DeoxoDS and DS in Arabidopsis

Compound	Rt^*b*^ on HPLC	RRt^*c*^ on GC	Prominent ions	Endogenous amount^*d*^
Arabidopsis				
6-DeoxoDS^*a*^	34–37	0.721	496 (M^+^, 26), 356 (1), 342 (1), 327 (1), 273 (44), 153 (67), 124 (100)	0.13
DS^*a*^	14–15	1.002	510 (M^+^, 22), 495 (16), 411 (16), 387 (15), 355/356 (23), 327 (96), 287 (9), 153 (69), 124 (100)	0.01
Authentic				
6-DeoxoDS^*a*^	34–37	0.721	496 (M^+^, 24), 356 (1), 342 (1), 327 (1), 273 (46), 153 (87), 124 (100)	—
DS^a^	14–15	1.002	510 (M^+^,25), 495 (13), 411 (12), 387 (18), 355/356 (20), 327 (95), 287 (10), 153 (72), 124 (100)	—

^*a*^The sample was analysed as bismethanboronate.

^*b*^Retention time.

^*c*^Relative retention time to CS (30.10 min).

^*d*^Amount is denoted as ng g^−1^ fresh weight.

6-DeoxoDS is thought to be a direct biosynthetic precursor of DS in Arabidopsis (Lee *et al.*, 2010). To confirm this, metabolism of 6-deoxoDS was examined using a crude enzyme solution prepared from Arabidopsis. Following enzyme assay, products were purified by reversed-phase HPLC, and the obtained HPLC fractions were tested in rice lamina inclination assays. In addition to HPLC fraction 34–37 for 6-deoxoDS, HPLC fractions 14–15, 19–21, and 40–42 showed biological activities. The HPLC fractions were derivatized to BMBs and analysed by capillary GC-MS. As summarized in Table 2, DS, CS, and 6-deoxoCS were present in HPLC fractions 14–15, 19–21, and 40–42, respectively. Coupled with identification of CS as an enzyme product of DS, this strongly suggests that partial biosynthetic sequences, 6-deoxoDS→DS→CS and 6-deoxoDS→6-deoxoCS→CS, are operating in Arabidopsis plants.

**Table 2.** *In vitro* conversion of 6-deoxoDS and DS in enzyme solution obtained from Arabidopsis

Substrate	Product	Rt^*b*^ on HPLC	RRt^*c*^ on GC	Prominent ions
6-DeoxoDS	6-DeoxoCS^*a*^	40–42	0.705	498 (M^+^, 35), 483 (15), 332 (4), 273 (100), 213 (4), 155 (37)
	DS^*a*^	14–15	1.002	510 (M^+^, 26), 495 (15), 411 (15), 387 (17), 355/356 (20), 327 (97), 287 (10), 153 (72), 124 (100)
	CS^*a*^	19–21	1	512 (M^+^, 81), 358 (35), 327 (10), 287 (29), 155 (100)
DS	CS^*a*^	19–21	1	512 (M^+^, 80), 358 (33), 327 (12), 287 (32), 155 (100)

^*a*^The sample was analysed as bismethanboronate.

^*b*^Retention time.

^*c*^Relative retention time to CS (30.10 min).

### DWF1 exhibits BR C-24 reductase activity in Arabidopsis

**Fig. 2.**
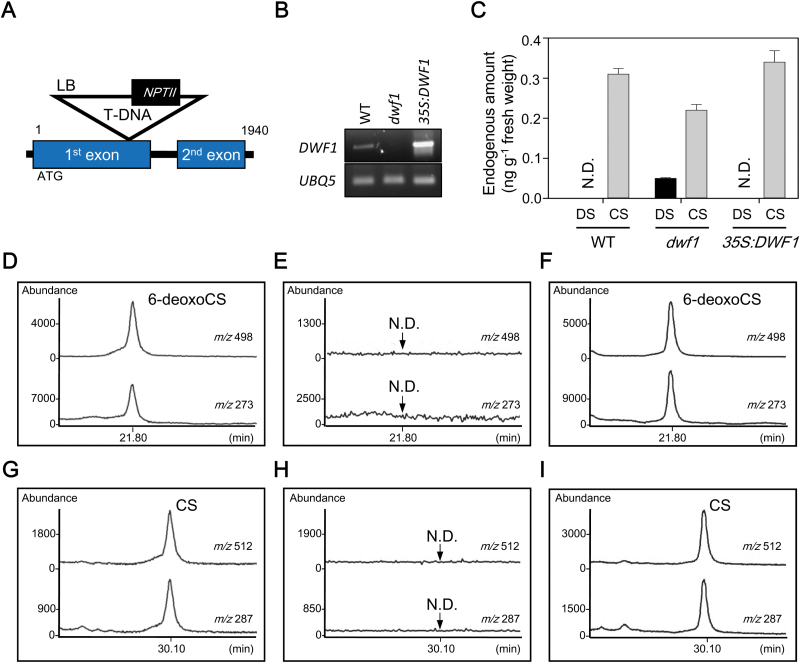
Biochemical analysis of DWF1 enzyme activity as a BR C-24 reductase in Arabidopsis. (A) Schematic diagram of T-DNA insertion in *dwf1* mutant. Exons and intron are indicated by boxes and a line, respectively. (B) Semi-qRT-PCR analysis of *DWF1* expression in the 40-day-old *dwf1* and *35S:DWF1* plants. (C) Endogenous amounts of DS and CS in *dwf1*, *35S:DWF1*, and wild-type (50 g each), which represent the average value obtained from two independent quantitative analyses (+SE). DS was quantified by a GC-SIM-based calibration curve using molecular ion at *m*/*z* 510. CS was quantified via GC-SIM using an internal standard (D_6_-labeled CS). (D–I) *In vitro* enzymatic conversion of 6-deoxoDS to 6-deoxoCS and DS to CS in wild-type (D, G), *dwf1* (E, H), and *35S:DWF1* (F, I). In GC-SIM analysis, ions at *m*/*z* 498 (M^+^) and *m*/*z* 273 were monitored for 6-deoxoCS-BMB. Ions at *m*/*z* 512 (M^*+*^) and *m*/*z* 287 were monitored for CS-BMB. N.D., not detected.

Arabidopsis *DWF1* loss-of-function mutant *dwf1* was selected from SIGnAL (http://signal.salk.edu/cgi-bin/tdnaexpress) mutant pools. Sequence analysis of genomic DNA flanking the T-DNA insertion sites revealed that *dwf1* contained a T-DNA insertion at the first exon in chromosome 3 (Fig. 2A). Semi-qRT-PCR analysis using RNA isolated from the homozygous mutant and from the wild-type (Col-0) showed that *dwf1* was a null allele (Fig. 2B; Supplementary Fig. S2A).

DWF1 is a sterol C-24 reductase that catalyses C-24 reduction of 24-MCHR to CR in Arabidopsis plants (Choe *et al.*, 1999). In *dwf1* mutants, endogenous levels of 24-MCHR are greatly increased compared with wild-type (Table 3). On the other hand, endogenous levels of CR are significantly reduced compared with those in wild-type. Therefore, loss-of-function of DWF1 as a sterol C-24 reductase was biochemically verified in the *dwf1* mutant.

**Table 3.** Endogenous amount of 24-MCHR and CR in *dwf1* and wild-type (Col-0) Arabidopsis

Compound	Wild-type (Col-0)		*dwf1*	
	Experiment 1	Experiment 2	Experiment 1	Experiment 2
24-MCHR (µg g^−1^ fresh weight)	5260	5560	15 591	13 940
CR (µg g^−1^ fresh weight)	44 369	43 990	668	673

Conversion of 6-deoxoDS to 6-deoxoCS and of DS to CS occurs via the same C-24 reduction mechanism as conversion of 24-MCHR to CR, implying that DWF1 can also mediate C-24 reduction of 6-deoxoDS to 6-deoxoCS and DS to CS in Arabidopsis. Endogenous levels of DS and CS were compared among wild-type (Col-0), *dwf1*, and *35S:DWF1* (*DWF1* overexpression line; Fig. 2B; Supplementary Fig. S2A), each with 50 g of Arabidopsis plants. As shown in Fig. 2C, Col-0 contained 0.31 ng g^−1^ fresh weight CS, but no detectable amount of DS. In *35S:DWF1*, CS was slightly increased (0.34 ng g^−1^ fresh weight) compared with Col-0, but no DS was detected, indicating that overexpression of *DWF1* can generate increased CS in *35S:DWF1*. In *dwf1*, CS was reduced (0.22 ng g^−1^ fresh weight) relative to Col-0, and 0.05 ng g^−1^ fresh weight of DS was detected. To demonstrate BR C-24 reductase activity of DWF1, conversion of 6-deoxoDS to 6-deoxoCS and DS to CS was examined in enzyme solutions prepared from *dwf1* and *35S:DWF1* plants. In GC-SIM analysis, C-24 reduction of 6-deoxoDS to 6-deoxoCS and DS to CS was shown in the enzyme solution prepared from *35S:DWF1*, but not in the enzyme solution prepared from *dwf1*, compared with those in wild-type (Fig. 2D–I). This suggests that DS accumulates in *dwf1* due to loss-of-function in the enzymatic activity for conversion of 6-deoxoDS to 6-deoxoCS and DS to CS in the mutant. In other words, DWF1 exhibits a BR C-24 reductase activity to convert 6-deoxoDS to 6-deoxoCS and DS to CS as well as a sterol C-24 reductase in Arabidopsis.

### Overexpression of *DWF1* enhances growth and development in Arabidopsis

**Fig. 3.**
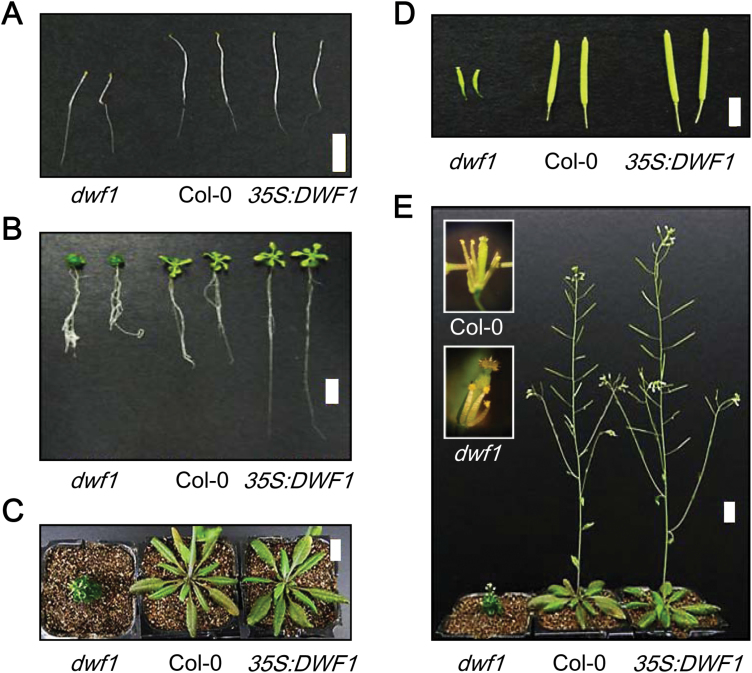
Phenotype of Arabidopsis wild-type (Col-0), *dwf1*, and *35S:DWF1* plants. (A) Comparison of 5-day-old dark-grown seedlings and (B) 7-day-old light-grown seedlings on MS media for *dwf1*, *35S:DWF1*, and wild-type. (C) Rosette leaves of 27-day-old *dwf1*, *35S:DWF1*, and wild-type plants. (D) Siliques and (E) adult phenotype of the *dwf1*, *35S:DWF1*, and wild-type. Scale bar=1 cm.

Abnormal growth and development in *dwf1* have been reported by several authors (Takahashi *et al.*, 1995, Klahre *et al.*, 1998, Choe *et al.*, 1999). As shown in Fig. 3, similar abnormalities were found in *dwf1* in this study. Phenotypic alterations in *35S:DWF1* were investigated over the entire developmental growth stage of transgenic plants. In the dark, *35S:DWF1* exhibited almost no differences in seedling growth compared with wild-type seedlings (Fig. 3A). Under long-day conditions (16 h light and 8 h dark), however, 12-day-old *35S:DWF1* seedlings showed more developed leaves and longer roots compared with wild-type plants (Fig. 3B). In the rosette plant stage, *35S:DWF1* showed similar sized leaves, but their shapes were more expanded than those in wild-type plants (Fig. 3C). In intact plants, *35S:DWF1* showed stems with slightly increased fluorescence as well as an increased number of siliques (Fig. 3D, E). These phenotypic alterations in *35S:DWF1* imply that overexpression of *DWF1* yields positive effects on growth and development of Arabidopsis under light conditions.

### DWF1 is important for biosynthesis of active BRs in Arabidopsis

**Fig. 4.**
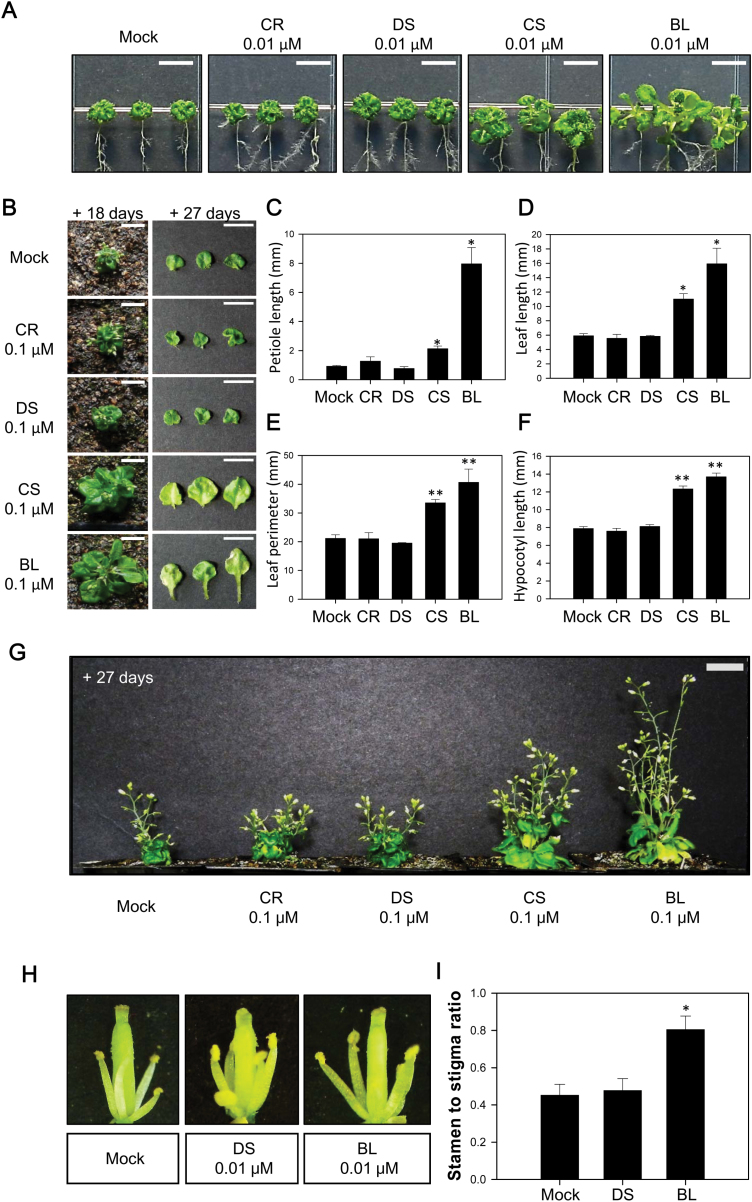
Reversal of abnormalities in *dwf1* through application of BRs. (A) Restoration of shoot growth in 7-day-old *dwf1* seedlings after application of 0.01 µM CR, DS, CS, and BL. Scale bar=1 cm. (B–E) Restoration of shoot growth in adult *dwf1* plants after application of 0.1 µM CR, DS, CS, and BL. BRs were sprayed 3–5 times a day onto *dwf1* for 27 d at an interval of 3 d. A representative result from six individual *dwf1* adult plants is shown in (B). Comparison of petiole length (C), leaf length (D), and leaf perimeter (E) after application of 0.01 µM CR, DS, CS, and BL. Each column represents the mean (+SE) of the individual measurements (*n*>20). (F) Restoration of hypocotyl growth in *dwf1* mutant seedlings after application of 0.1 µM CR, DS, CS and BL. Hypocotyl length was measured after 7 d. Each column represents the mean (+SE) of the individual measurements (*n*>20). Asterisks indicate the statistical significance by Student’s *t*-test. *P<0.05, **P<0.01 compared with Mock control. (G) Restoration of abnormal stem growth in *dwf1* adult plants after application of 0.1µM CR, DS, CS, and BL. BRs were sprayed 3–5 times a day onto *dwf1* for 27 d at an interval of 3 d. A representative result from six individual *dwf1* intact plants is shown. (H, I) Restoration of stamen development of *dwf1* after 0.01 µM DS and BL treatment. After artificial fertilization, BRs were sprayed 3–5 times onto *dwf1* flower every day. A representative result from five individual *dwf1* flowers is shown in (H). Hormonal effects on phenotypic rescue of less-developed stamen in *dwf1* are presented as stigma to stamen length ratio (I). Each column represents the mean (+SE) of the individual measurements (*n*=5).

Restoration of phenotypic abnormalities in *dwf1* was examined by application of CR, DS, CS, and BL. In light-grown seedlings and rosette plants, application of CR and DS did not improve abnormal leaf size, leaf shape or petiole growth, while application of CS and BL almost completely restored leaf size, leaf shape and petiole length to that observed in wild-type plants (Fig. 4A–E). In dark-grown seedlings, application of CS and BL greatly increased hypocotyl length compared with CR and DS application (Fig. 4F; Supplementary Fig. S3A). In intact plants, abnormalities found in *dwf1* such as unexpanded and less-developed dark green rosette leaves, reduced fluorescence in stems, and less-developed stamen were not restored by application of CR and DS. Abnormal stem growth and development of rosette leaves and reproductive organs in *dwf1* were clearly reversed by application of CS and BL (Fig. 4G–I). These findings indicate that abnormalities shown in *dwf1* are likely caused by insufficient endogenous levels of CS and BL.

**Fig. 5.**
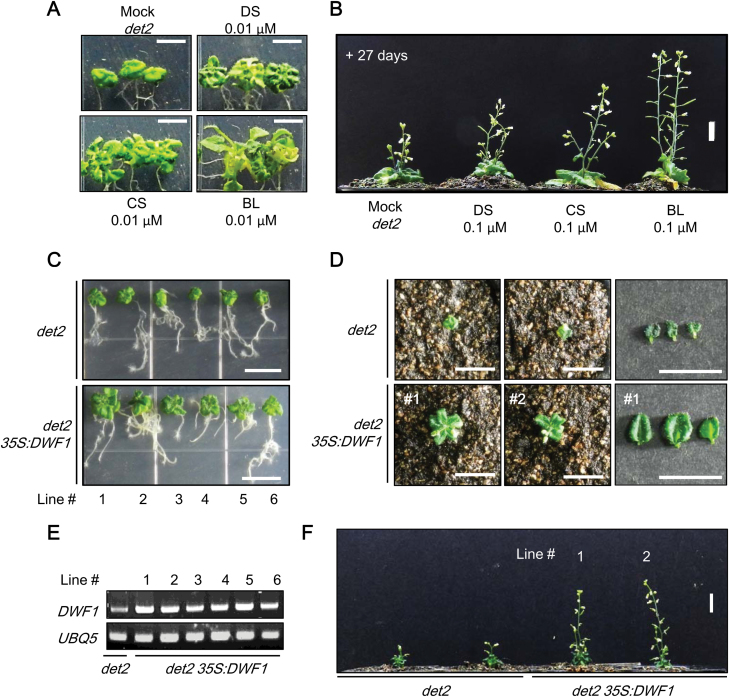
Reversal of abnormalities in *det2* via application of BRs and overexpression of *DWF1*. (A) Restoration of shoot growth in *det2* mutant seedlings after application of 0.01 µM DS, CS, and BL. Seedlings were grown on vertically oriented MS plates for 7 d in the presence or absence of BRs. Scale bar=1 cm. (B) Restoration of shoot growth in *det2* adult plants after application of 0.1 µM DS, CS, and BL. BRs were sprayed 3–5 times a day onto *det2* for 27 d at an interval of 3 d. A representative result from 10 individual *det2* adult plants is shown in (B). (C–F) Reversal of the *det2* abnormal phenotype in *det2 35S:DWF1* at the seedling (C), rosette (D), and intact plant (F) stages. Expression levels of *DWF1* in each *det2 35S:DWF1* transgenic line were confirmed by semi-qRT-PCR (E).

Restoration of abnormalities in a BR-deficient mutant, *det2*, was tested by application of BRs. Exogenously applied DS as well as CS and BL successfully rescued abnormalities in *det2* (Fig. 5A, B; Supplementary Fig. S3B). Next, *DWF1* was overexpressed in *det2*, and the recovery of the phenotype in *det2 35S:DWF1* was examined. As shown in Fig. 5C–F, abnormalities in *det2* were clearly reversed in *det2 35S:DWF1* at the seedling, rosette, and intact plant stages, which demonstrates a significant role of DWF1 in BR biosynthesis in Arabidopsis plants.

### *DWF1* expression is down-regulated by BR signaling in Arabidopsis

**Fig. 6.**
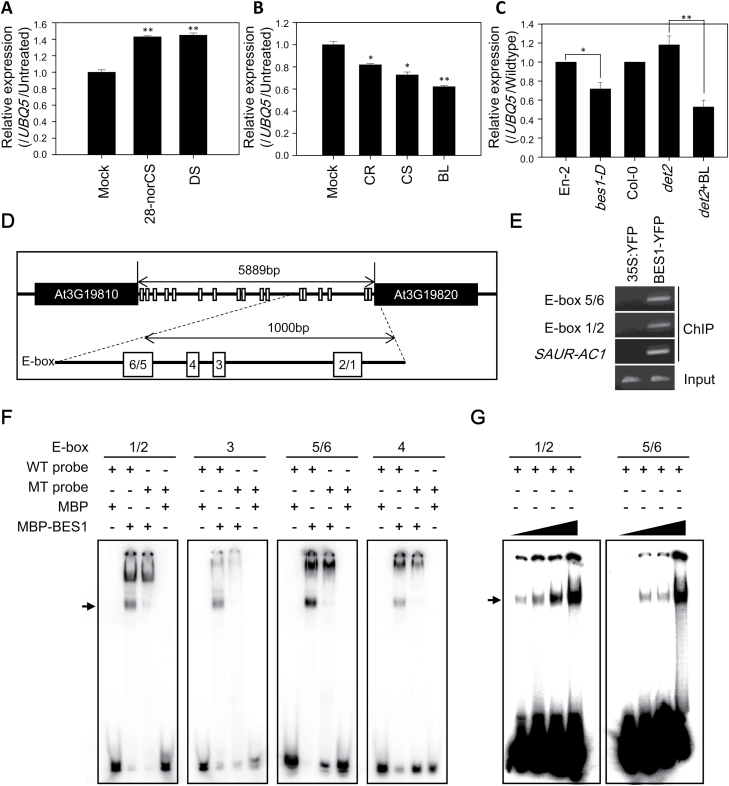
Feedback regulation of *DWF1* by BR signaling via BES1 in Arabidopsis. (A) Comparison of *DWF1* expression by application of 28-norCS and DS. (B) Comparison of *DWF1* expression by application of CR, CS, and BL. Seedlings grown in 0.5× MS medium for 7 d were transferred to DW containing 0.1 µM BRs and incubated for 2 h. (C) Expression of *DWF1* in BR-deficient (*det2*) and dominant (*bes1-D*) mutants. Data are presented as a relative value (mean ±SE) relative to Mock control (A, B) and wild-type (C). Asterisks indicate the statistical significance by Student’s *t*-test. *P<0.05, **P<0.01 compared with mock or wild-type. (D) Schematic diagram of *DWF1* promoter region. White rectangles indicate E-boxes (CANNTG) on the intergenic region. The six E-boxes were denoted as E-box 1 to E-box 6. E-box 1/2 and 5/6 contain two overlapped E-boxes. (E) ChIP assay results for binding of BES1 to *DWF1* promoter *in vivo* (lanes 1 and 2; Input, lanes 3–8; ChIP with anti-YFP antibody prepared from 10-day-old light-grown *35S:YFP* (negative control) and *BES1-YFP* transgenic seedlings). ChIP-PCR was performed with primers from indicated E-box positions at the *DWF1* promoter and *SAUR-AC1* (positive control). Each assay was repeated three times. (F, G) EMSA results for binding of BES1 to *DWF1* promoter *in vitro* (F). ^32^P-labeled *DWF1* probes were incubated with or without MBP-tagged BES1 protein. The absence or presence of MBP-tagged BES1 protein is indicated by – or +. MBP proteins and mutated probes (MT probes) were used as a negative control (G). ^32^P-labeled *DWF1* probes were incubated at an increasing concentration (0.5, 1, 1.5, and 2 µg) of MBP-tagged BES1 protein.

Molecular regulation of *DWF1* expression in BR biosynthesis was examined in Arabidopsis. Exogenously applied DS and 28-norCS as a potent biosynthetic precursor of DS increased *DWF1* expression (Fig. 6A), implying that *DWF1* expression is up-regulated by the substrate and its biosynthetic precursor. In contrast, exogenously applied CR, CS, and BL inhibited expression of *DWF1* (Fig. 6B), which suggests that *DWF1* expression is down-regulated by the products. In a BR-deficient mutant (*det2*), expression of *DWF1* was increased compared with that in wild-type (Col-0), but this increased *DWF1* expression was greatly reduced when BL was applied to *det2* (Fig. 6C). Compared with wild-type (En-2), expression of *DWF1* was inhibited in the BR-dominant mutant, *bes1-D* (Fig. 6C), while expression of *DWF1* was not changed in the gain-of function mutant of the BZR1, *bzr1-1D* (Supplementary Fig. S4). These results suggest that feedback regulation of *DWF1* by the products mainly occurs through BR signaling via a transcription factor, BES1 in Arabidopsis.

Seventeen *cis*-regulatory elements (E-boxes, CANNTG) for BES1 binding sites were located on the intergenic region of *DWF1* (at 5889 bp) in the Arabidopsis genome (Fig. 6D). In particular, six E-boxes were present in the region 1000-bp upstream from the start codon of *DWF1*, a putative promoter region for *DWF1*. The six E-boxes were denoted E-box 1 to E-box 6, which were respectively nearest to and farthest from the start codon of *DWF1*. Using BES1–YFP transgenic plants, a ChIP assay was carried out. As shown in Fig. 6E, BES1–YFP proteins successfully bind to both E-box 1/2 and E-box 5/6 in Arabidopsis plants (Supplementary Fig. S2B). Maltose binding protein (MBP)–BES1 was obtained from *E. coli* using a construct in which MBP was fused to the N-terminus of full-length BES1, and an EMSA was also conducted. As shown in Fig. 6F, MBP, used as a negative control, was not bound to six E-boxes, but MBP–BES1 was directly bound to the E-boxes (Fig. 6F; Supplementary Fig. S2C). Two overlapped probes in particular containing E-box 1/2 and E-box 5/6 were strongly bound to MBP–BES1 in a concentration-dependent manner (Fig. 6G; Supplementary Fig. S2D). The binding of MBP–BES1 was diminished by mutation of E-boxes (CANNTG to AAAAAA) and excess unlabeled probes (Supplementary Figs S2E and S5). These results demonstrate that BES1 binds to the E-boxes on the promoter region of *DWF1* both *in vitro* and *in vivo*.

## Discussion

**Fig. 7.**
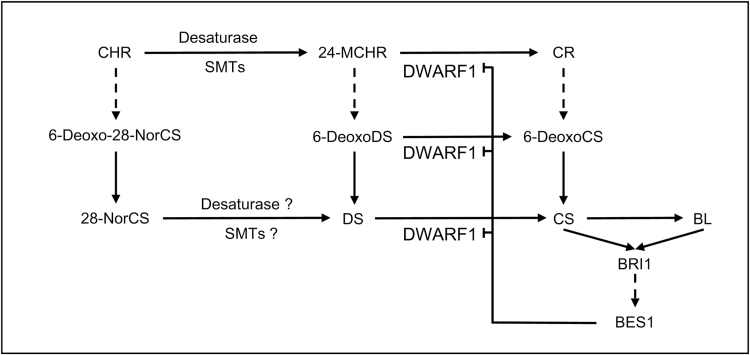
Connections of three BR biosynthetic pathways that function in Arabidopsis. Three biosynthetic pathways are funneled into CS. *DWF1* is feedback-regulated through BR signaling via a transcription factor, BES1, in the plant. Solid arrows indicate a single biosynthetic step. Dotted arrows indicate multiple biosynthetic steps. SMT, sterol methyltransferase.

In many plants, the biosynthetic end products of C_27_-, C_28_-, and C_29_-BRs co-exist, suggesting that multiple biosynthetic pathways function within the plants (Fujioka *et al.*, 1997; Yokota, 1997; Sakurai, 1999; Bajguz and Tretyn, 2003; Joo *et al.*, 2009). In tomato plants, endogenous levels of a C_27_-BR, 28-norCS, are comparable to that of CS (Yokota *et al.*, 1997). *In vitro* conversion experiments using enzyme solutions prepared from tomato plants revealed that 28-norCS is biosynthesized from cholesterol (CHR) via the same late C-6 oxidation pathway as has been established in CS and BL biosynthesis (Kim *et al.*, 2004*b*). In Arabidopsis, endogenous 28-norCS can also be biosynthesized from CHR via the late C-6 oxidation pathway. However, the early C-6 oxidation pathway for 28-norCS is interrupted between 6-oxocholestanol (6-oxoCHN) and 28-norTE, as it is intermediated by 28-norCT (Joo *et al.*, 2012). In the biosynthetic pathway of 28-norCS, involvement of DET2, CYP90B1, CYP85A1, and CYP85A2 has been demonstrated. Although the presence of biosynthetic sequences, such as 22-hydroxy-CHR→22-hydroxy-cholesta-4-en-3-one→22-hydroxy-cholesta-3-one→6-deoxo-28-norCT and 3-*epi*-6-deoxo-28-norCT, and the involvement of CYP90C1 and CYP90D1 for C-23 hydroxylation of 22-hydroxy-cholesta-3-one, 6-deoxo-28-norCT, and 3-*epi*-6-deoxo-28-norCT to 6-deoxo-28-norTE, 3-dehydro-6-deoxo-28-norTE, and 6-deoxo-28-norTY, respectively, have not yet been demonstrated, these suggest that 28-norCS is biosynthesized through a cholestanol (CHN)-independent pathway via the same biosynthetic reactions that have been established in CN-independent pathways for the biosynthesis of CS and BL in Arabidopsis (Supplementary Fig. S1). Enzyme solutions prepared from both tomato and Arabidopsis successfully mediate conversion of 28-norCS to CS, providing that biosynthesis of 28-norCS, a C_27_-BR, biosynthesis is an alternative route to produce a biologically active BR, CS, in both plants (Fig. 7, Kim *et al.*, 2004*b*; Joo *et al.*, 2012).

In the current study, we identified 6-deoxoDS in Arabidopsis. Coupled with the presence of DS and 24-MCHR (Fujita *et al.*, 2006), identification of 6-deoxoDS suggests that 24-methylene BR biosynthesis took place in Arabidopsis plants. CYP90B1 can catalyse 22-hydroxylation of 24-MCHR to 22-hydroxy-24-MCHR (Fujita *et al.*, 2006). Previous results from our group demonstrated that C-6 oxidation of 6-deoxoDS to DS is primarily mediated by CYP85A2 (Lee *et al.*, 2010). Although the presence of biosynthetic sequences from 22-hydroxy-24-MCHR to 6-deoxoDS and the involvement of CYP90B1, CYP90C1/D1, and DET2 in 24-methylene BR biosynthesis have not yet been established, the results of this study imply that 24-methylene BRs are biosynthesized via a similar biosynthetic route to that in the biosynthesis of 28-norCS and CS. DL was not found even in large quantities of Arabidopsis. In addition, enzyme prepared from Arabidopsis was unable to catalyse the conversion of DS to DL. Therefore, the end product of 24-methylene BR biosynthesis in Arabidopsis plants seems to be DS rather than DL.

The crude enzyme solution prepared from *P. vulgaris* and Arabidopsis successfully mediated conversion of 6-deoxoDS to CS via DS. In the study where we demonstrated this conversion, we also demonstrated conversion of 6-deoxoDS to CS via 6-deoxoCS in Arabidopsis plants. This evidence suggests that biosynthesis of 24-methylene BRs and 24-methyl BRs involves a connection between 6-deoxoDS and 6-deoxoCS and between DS and CS in Arabidopsis. We recently demonstrated in Arabidopsis that biosynthetic precursors for 28-norCS such as 28-norTE, 28-nor-3-dehydroTE, and 28-norTY can be converted into biosynthetic precursors for CS such as TE, 3-dehydroTE and TY, respectively (Joo *et al.*, 2012). Therefore, the biosynthetic pathways for 28-norCS, DS and CS are likely to be connected in Arabidopsis not only between end products, but also between their biosynthetic precursors. With complicated connections, three biosynthetic pathways for BRs are funneled into CS to maintain biologically active BRs, CS and BL in plants (Fig. 7).

In Arabidopsis, *dwf1*, *diminuto* (*dim*), and *cabbage1* (*cbb1*) alleles are associated with typical BR-deficient phenotypes such as abnormal root and leaf development, reduced stem elongation (dwarfism), failed stamen formation, reduced silique and seed formation, and delayed senescence. All mutants exhibit accumulation of side chain unsaturated phytosterols at C-24, such as 24-MCHR and isofucosterol, whereas they show reductions in C-24 saturated phytosterols, such as CR and sitosterol (Takahashi *et al.*, 1995, Klahre *et al.*, 1998, Choe *et al.*, 1999). Similarly, a pea *lkb* mutant shows the same phenotypes as the BR-deficient mutant, while 24-MCHR and isofucosterol accumulate to levels comparable to those in *dim*, suggesting that the *LKB* gene is an ortholog of *DWF1* (Nomura *et al.*, 1997; Nomura *et al.*, 1999). Accompanied by feeding experiments using isotope-labeled substrates, these results suggest that in plants, DWF1 catalyses conversion of 24-MCHR and isofucosterol to CR and sitosterol, respectively, as a sterol C-24 reductase (Klahre *et al.*, 1998; Choe *et al.*, 1999). Nevertheless, exogenous application of CR cannot reverse abnormalities in *dwf1* to their original states in wild-type plants, while the application of CS, BL, and their biosynthetic precursors can reverse these abnormalities (Klahre *et al.*, 1998). Although the reason why exogenously applied CR could not restore *dwf1* phenotype is still unknown, the conversion of campesterol to CS and BL seems to be inefficient or ineffective for rescuing the *dwf1* phenotype. In this study, we also found that abnormalities in *dwf1* cannot be rescued by CR, but this can be achieved by CS and BL (Fig. 4). Therefore, the phenotype found in *dwf1* is likely to originate from deficiency of active BRs and not from phytosterol deficiency in Arabidopsis.

Based on GC-SIM quantification with 30 kg of Arabidopsis, endogenous amounts of DS were calculated to be approximately 0.01 ng g^−1^ fresh weight in Arabidopsis (Table 1). When 50 g of Arabidopsis was used for quantitative analysis, endogenous levels of DS were not detectable on GC-SIM analysis, which resulted in a lack of DS in wild-type plants and in *35S:DWF1* mutants (Fig. 2C). However, approximately 0.05 ng g^−1^ fresh weight of DS was detected in the *dwf1* mutant. In contrast, an endogenous amount of CS at 0.31 ng g^−1^ fresh weight in wild-type was reduced to 0.22 ng g^−1^ fresh weight in the *dwf1* mutant. Therefore, defects in *DWF1* caused accumulation of DS and reduction of CS via blockage of conversion of DS to CS in the *dwf1* mutant. We previously reported that an approximately 20% reduction in CS can cause severe abnormalities in *cyp85a2* (Kim *et al.*, 2005*b*), suggesting that the abnormal phenotype in *dwf1* is caused by a 29% reduction in CS in the mutant. In *35S:DWF1*, DS was not detected by GC-SIM analysis, but endogenous levels of CS in *35S:DWF1* were slightly increased at 0.34 ng g^−1^ fresh weight. This may have enhanced the formation of BL, the most biologically active BR in Arabidopsis, resulting in slightly increased stem elongation, silique formation, and altered root development in the *35S:DWF1* mutant. Collectively, changes in endogenous CS level seem to induce phenotypic alternations in *dwf1* and *35S:DWF1*, which suggests that 24-methylene BR biosynthesis to generate CS catalysed by DWF1 is required for homeostasis of active BRs to control the growth and development of Arabidopsis plants.

In Arabidopsis, endogenous CS is extremely reduced (to <10%) in BR-deficient dwarf mutants such as *dwf4*, *det2*, and *cyp85a1/cyp85a2* (Fujioka *et al.*, 1997; Choe *et al.*, 2001; Kim *et al.*, 2005*b*). In contrast, sterol-deficient dwarf mutants such as *smt1/2*, *dwf7*, and *dwf5* contain a fair amount (>50%) of CS, suggesting that the biosynthetic pathway to produce CS may be fairly operant with small amounts of sterols in sterol-deficient mutants (Choe *et al.*, 1999, 2000, 2001; Diener *et al.*, 2000; Carland *et al.*, 2010). In this study, we determined that a small amount of CR can be generated in *dwf1* in spite of DWF1 deficiency (Table 3). We also found that expression of biosynthetic genes for BRs such as *DWF4*, *CPD*, and *CYP85A2* is clearly up-regulated in *dwf1* (Supplementary Fig. S6). Therefore, endogenous CS (79% of CS in wild-type) can be effectively synthesized from a low level of CR by the activated biosynthesis of BRs in *dwf1*.

*BR-deficient dwarf2* (*brd2*), found in rice, is a homolog of Arabidopsis *dwf1* (Hong *et al.*, 2005). Like *dwf1*, *brd2* leads to accumulated 24-MCHR and DS, but reduced CR and CS. Exogenous application of DS to *brd2* exhibits activity similar to CS: sheath elongation, inhibited root growth, and inclined lamina, suggesting that DS is an alternative bioactive BR in rice. In this study, we also examined the extent to which application of DS, CS, and BL led to restoration in *dwf1* mutants. As shown in Fig. 4, application of DS led to little reversal of *dwf1* abnormalities. On the other hand, applied CS and BL led to complete reversal of abnormalities in Arabidopsis. This indicates that DS is not a biologically active BR, but rather a biosynthetic precursor to the generation of bioactive BRs, CS and BL in Arabidopsis.

DWF1 is a flavin adenine dinucleotide (FAD)-dependent oxidoreductase (Choe *et al.*, 1999). In CR biosynthesis, DWF1 exhibits enzyme activity for C-24 isomerization and for reduction of 24-MCHR (Klahre *et al.*, 1998). We previously proposed that C-24 methylation of 28-norCS to CS occurs via three steps: (i) desaturation of 28-norCS to form Δ^24^-28-norCS by a desaturase, (ii) *S*-adenosylmethionine-dependent methylation of Δ^24^-28-norCS to form DS by sterol methyltransferase1 (SMT1), and (iii) reduction of DS by NADPH to form CS by DWF1 (Joo *et al.*, 2012). In this study, we concretely demonstrate that the reduction of DS to CS is catalysed by DWF1. This reduction consists of isomerization and reduction, implying that DWF1 has both isomerase and reductase activities in the reduction in Arabidopsis. Successful conversion of 28-norCS to CS via DS indicates that DS can be biosynthesized from not only 24-MCHR, but also CHR via 28-norCS in Arabidopsis. Nevertheless, endogenous levels of DS are quite low in Arabidopsis, suggesting that DWF1 enzyme activity in the reduction of DS to CS can be high in plants. Expression of *DWF1* was enhanced by 28-norCS and DS (Fig. 6A). This feed-forward activation of *DWF1* by each 28-norCS and DS seems to be another reason that endogenous levels of DS are low in Arabidopsis.

Exogenously applied, biologically active BRs, primarily CS and BL, down-regulate expression of BR biosynthetic genes, but up-regulate BR signaling genes in Arabidopsis (Goda *et al.*, 2002; Tanaka *et al.*, 2005; Gruszka, 2013). This suggests that increased levels of active BRs trigger enhanced BR signaling towards down-regulation of biosynthetic genes in plants (Tanaka *et al.*, 2005). In the current study, we found *DWF1* expression to be down-regulated by active BRs (CS and BL) in Arabidopsis (Fig. 6B). In addition, application of BL led to decreased *DWF1* expression in *det2*, such that expression became much lower than that of the wild-type state (Fig. 6C). These findings indicate that *DWF1* expression was negatively regulated by endogenous levels of active BRs. *DWF1* expression was also down-regulated by CR. However, the down-regulation of *DWF1* by CR was weaker than that by CS and BL, suggesting that feedback regulation of *DWF1* is more effective in downstream BR biosynthesis rather than in upstream phytosterol biosynthesis with regard to maintaining physiological levels of active BRs in Arabidopsis.

In *bes1-D*, *DWF1* was down-regulated (Fig. 6C), implying that regulation of *DWF1* may have occurred through feedback in BR-induced signal transduction pathways in Arabidopsis (Fig. 7). In the intergenic region of *DWF1*, 17 E-boxes for BES1 binding sites were present, but no BR response element (BRRE) sequence for the BZR1 binding site were found. Furthermore, *DWF1* was not down-regulated in *bzr1-1D* (Supplementary Fig. S4). Although BZR1 can bind to E-boxes, this suggests that BES1 is a BR transcription factor that regulates *DWF1* transcription in plants. In fact, EMSA and ChIP showed direct binding of BES1 to E-boxes (Fig. 6E, F), more strongly to two overlapped E-boxes, 1/2 and 5/6, both located on the potent *DWF1* promoter region (Fig. 6F, G). Therefore, it is likely that down-regulation of *DWF1* occurs via direct binding of BES1 to the *DWF1* promoter in Arabidopsis. Feedback regulation of BR biosynthetic genes occurs mainly by BZR1 (Wang *et al.*, 2002, He *et al.*, 2005). From this viewpoint, this study provides a good example of BR biosynthetic genes also acting in feedback regulated by BES1 to maintain appropriate concentrations of endogenous BRs in Arabidopsis.

## Supplementary data

Supplementary data are available at *JXB* online.

Fig. S1. CN-independent (A) and CHN-independent (B) pathways for biosynthesis of BRs in Arabidopsis.

Fig. S2. Original images for all of the gels and blots presented in this report. (A) Fig. 2B, (B) Fig. 6E, (C) Fig. 6F, (D) Fig. 6G, (E) Supplementary Fig. S5.

Fig. S3. Hypocotyl length assay in 5-day-old seedlings of *dwf1* (A) and *det2* (B) mutant after application of 0.1 µM CR, DS, CS, and BL.

Fig. S4. Expression level of *DWF1* and *DWF4* in BR-dominant mutants *bes1-D* and *bzr1-1D.*

Fig. S5. Competitive EMSA results for binding of MBP-BES1 to *DWF1* promoter *in vitro*.

Fig. S6. Alternation of expression of BR biosynthetic genes in *dwf1* and wild-type (Col-0).

Table S1. Sequences of DNA probes and primers used in this study.

Supplementary_Figures_S1_S6_table_S1

## Abbreviations:

BL: brassinolide
BMB: bismethaneboronate
BR: brassinosteroid
CHN: cholestanol
CHR: cholesterol
CN: campestanol
CR: campesterol
CS: castasterone
CT: cathasterone
CYP: cytochrome P450
DL: dolicholide
DS: dolichosterone
DT: dehydroteasterone
24-MCHR: 24-methylenecholesterol
TE: teasterone
TY: typhasterol.
