# Supplementary material for: Function and molecular regulation of DWARF1 as a C-24 reductase in brassinosteroid biosynthesis in Arabidopsis
Source: J Exp Bot. 2018 Feb 8;69(8):1873–86. doi: 10.1093/jxb/ery038 (PMC6018864; doi:10.1093/jxb/ery038)
Supplement: Supplementary_Figures_S1_S6_table_S1 [file ery038_suppl_supplementary_figures_s1_s6_table_s1.pdf]

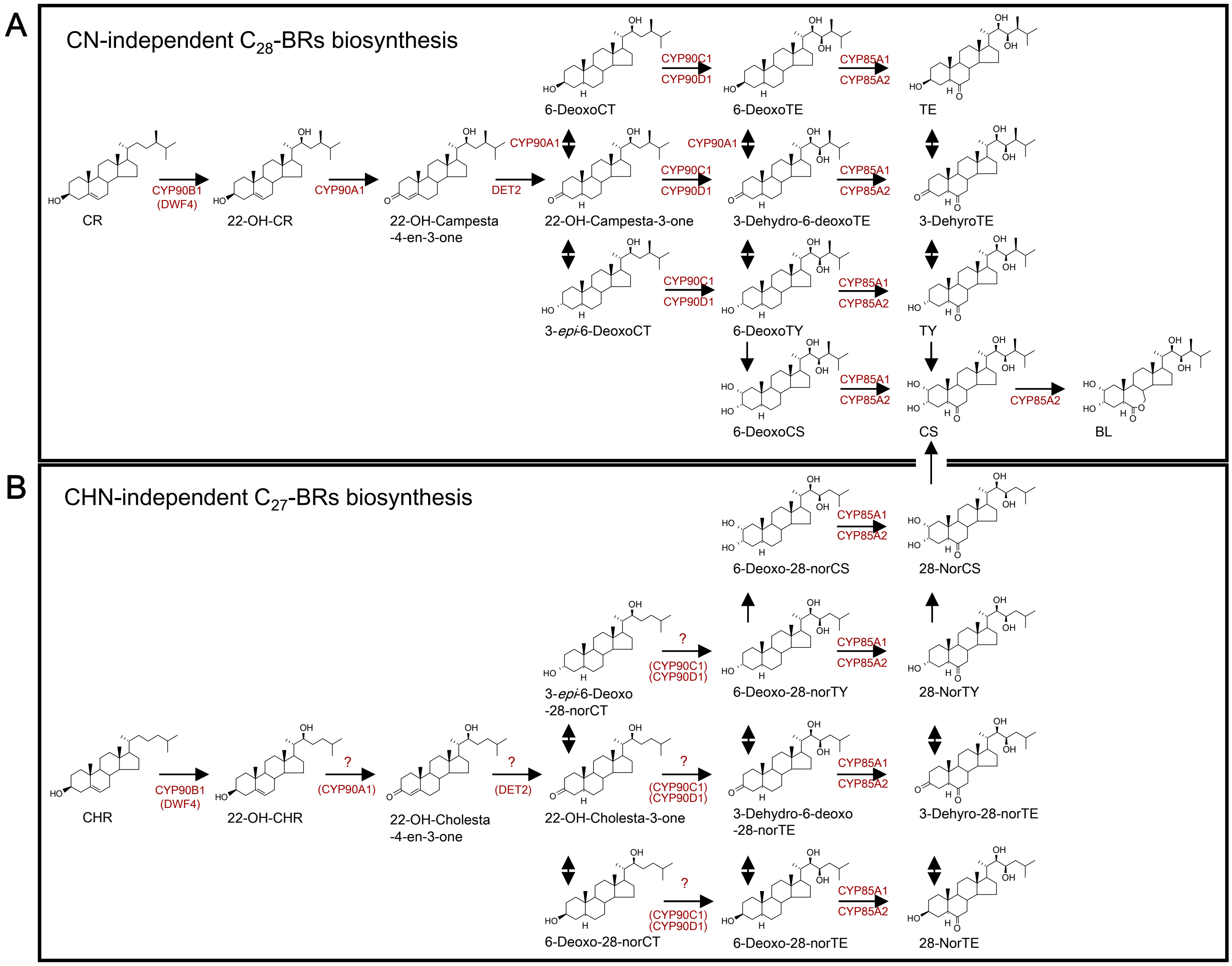

Supplementary Fig. S1 CN-independent (A) and CHN-independent (B) pathways for biosynthesis of BRs in Arabidopsis

A Fig. 2B

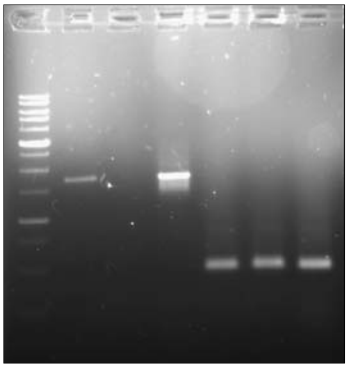

B Fig. 6E

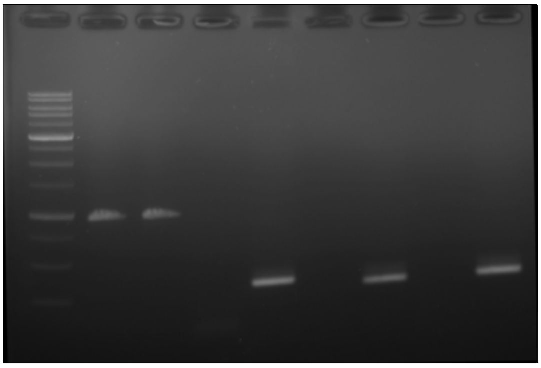

C Fig. 6F

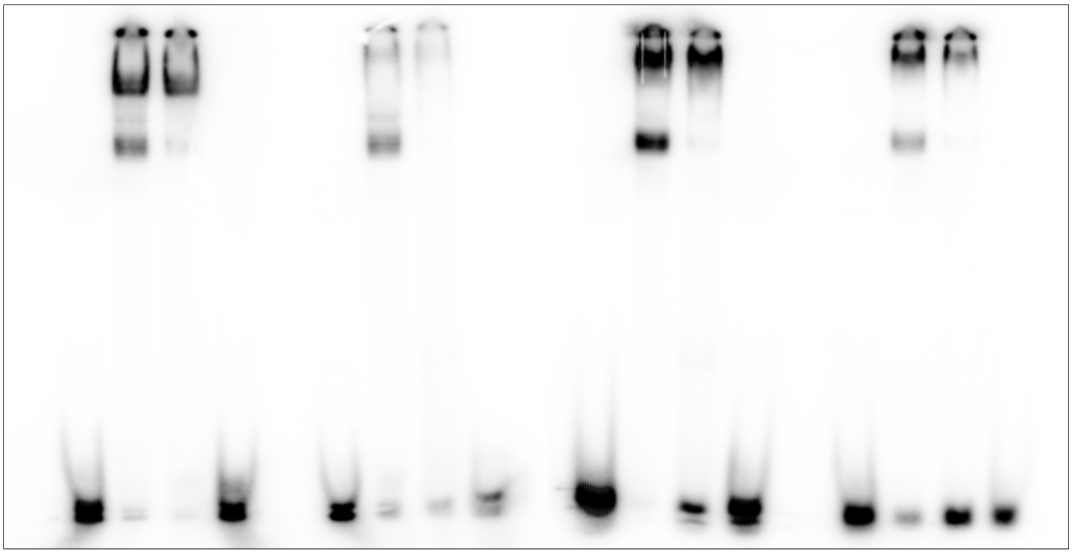

D Fig. 6G

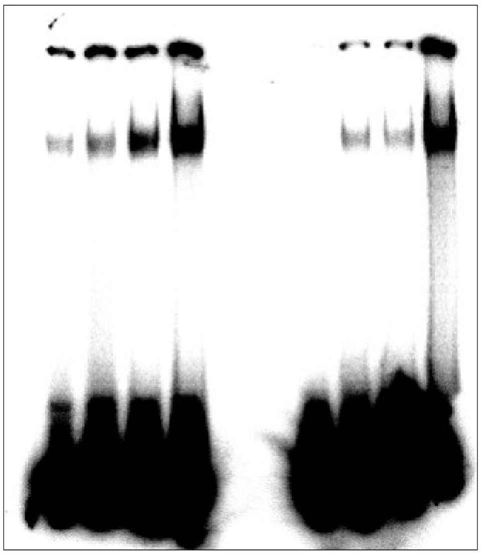

E Supplementary Fig. S4

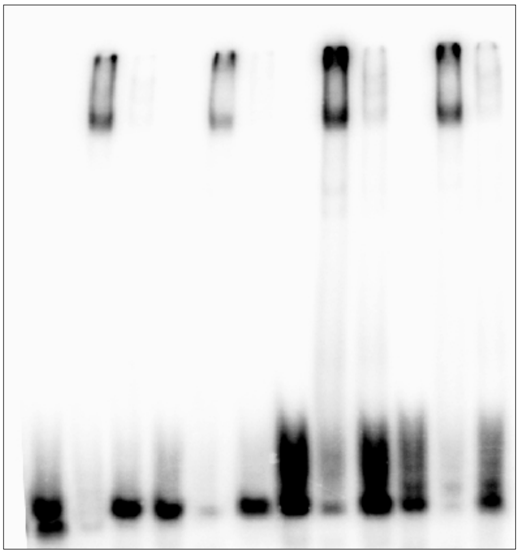

Supplementary Fig. S2 Original images for all of the gels and blots presented in this report. (A) Fig.2B, (B) Fig. 6E, (C) Fig. 6F, (D) Fig. 6G. (E) Supplementary Fig.S5

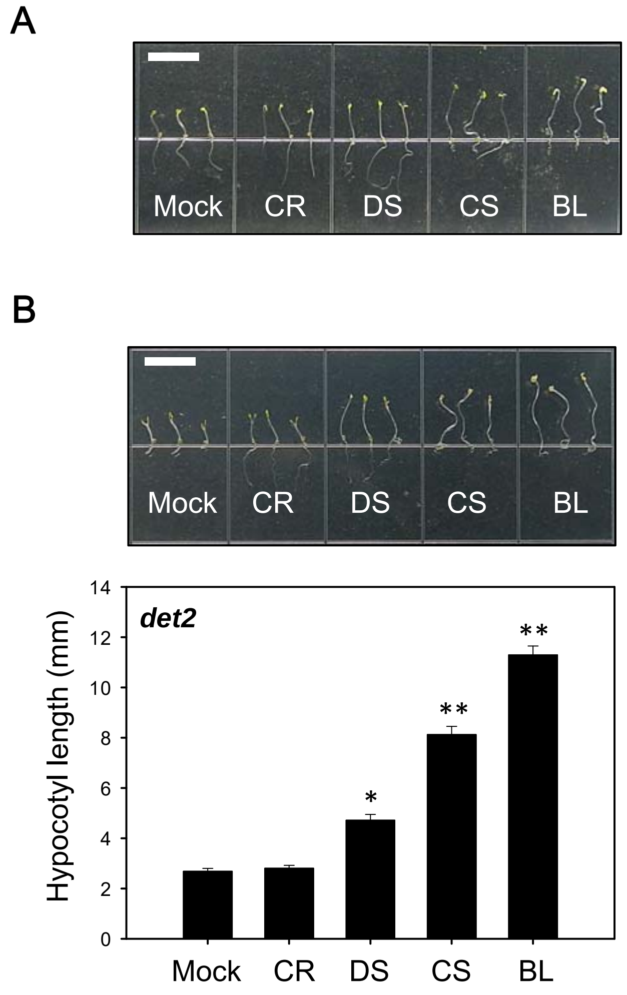

Supplementary Fig. S3 Hypocotyl length assay in 5-day-old seedlings of *dwf1* (A) and *det2* (B) mutant after application of 0.1 μM CR, DS, CS and BL.

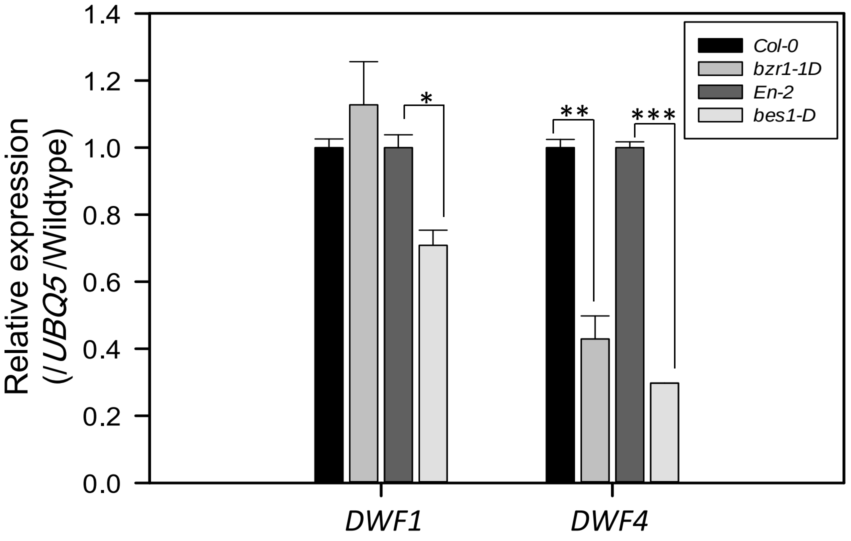

Supplementary Fig. S4 Expression level of *DWF1* and *DWF4* in BR-dominant mutants *bes1-D* and *bzr1-1D.*

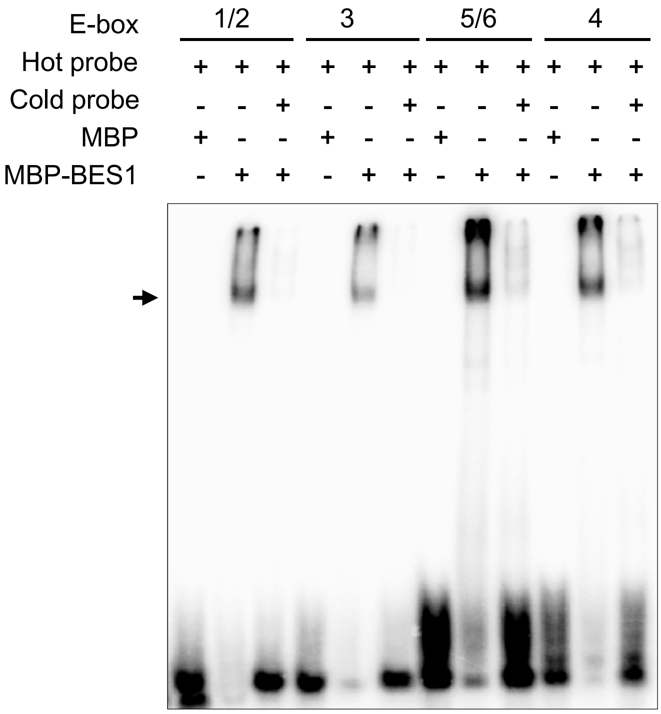

Supplementary Fig. S5 Competitive EMSA results for binding of MBP-BES1 to *DWF1* promoter in vitro.

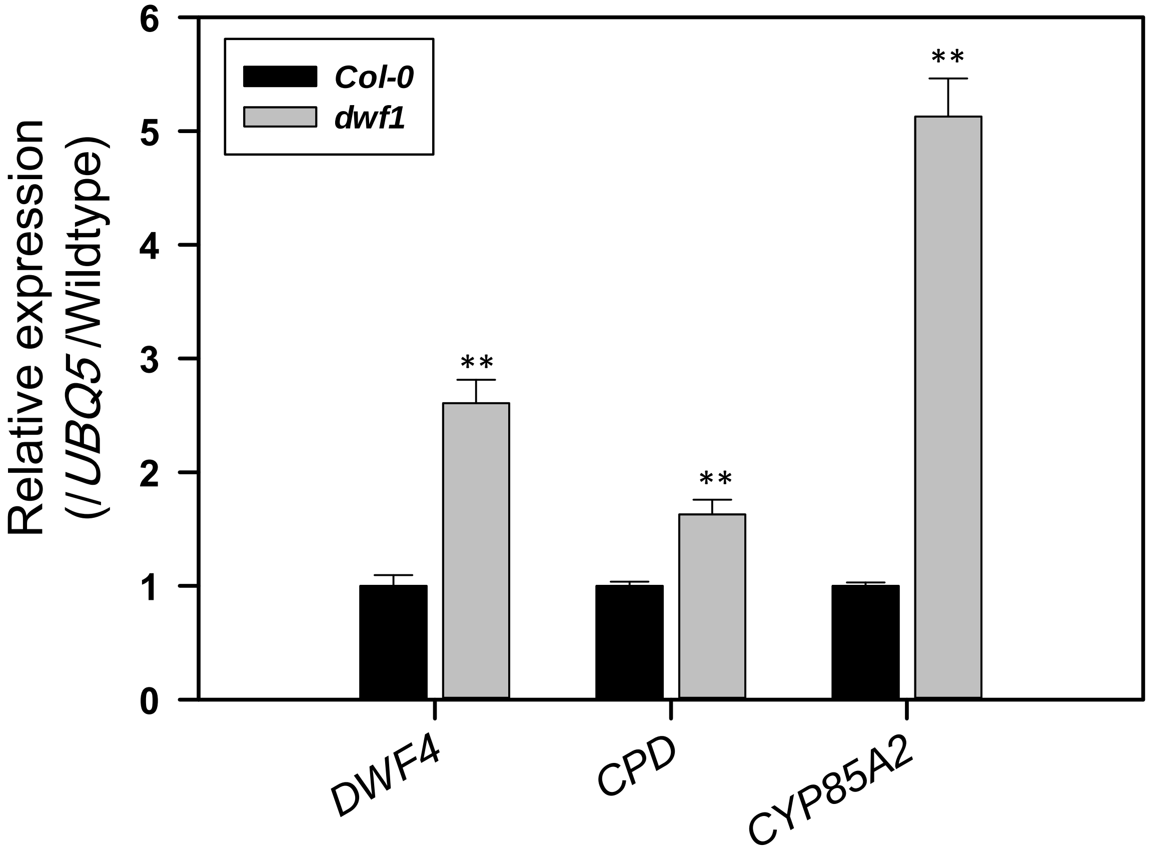

Supplementary Fig. S6 Alternation of expression of BR biosynthetic genes in *dwf1* and Wild-type (Col-0).

Supplementary table S1. Sequences of DNA probes and primers used in this study

**Name**		**Sequence**	**Usage**
SAUR-AC1-c	Forward	GCAGAAAGGAGTCAGTGATG	ChIP
	Reverse	AAGACAGGACCACATGATTTG	
DWF1 E-box1/2-c	Forward	GTGTAGATCTAAAGATTATAA	
	Reverse	GTCAACAAGGCCTAAGAAGT	
DWF1 E-box5/6-c	Forward	TGTCTAATTAAGCTCTAGGTTCGA	
	Reverse	TCGCAATTAAAAATTCGATGT	
DWF1 E-box1/2-e (WT)	Forward	TTTTTCTGATACACATGTGTGTATA	EMSA
	Reverse	TTTTTATACACACATGTGTATCAG	
DWF1 E-box3 (WT)	Forward	TTTTTATAAAGACAAATGCTCTCAC	
	Reverse	TTTTTGTGAGAGCATTTGTCTTTAT	
DWF1 E-box4 (WT)	Forward	TTTTTAAAAATGCAAGTGAAGCAGG	
	Reverse	TTTTTCCTGCTTCACTTGCATTTTT	
DWF1 E-box5/6-e (WT)	Forward	TTTTTATATAACACATGTGATTTAT	
	Reverse	TTTTTATAAATCACATGTGTTATAT	
DWF1 E-box1/2-e (MT)	Forward	TTTTTCTGATAAAAAAAAATGTATA	
	Reverse	TTTTTTATACATTTTTTTTTATCAG	
DWF1 E-box3-e (MT)	Forward	TTTTTATAAAGAAAAAAACTCTCAC	
	Reverse	TTTTGTGAGAGTTTTTTCTTTAT	
DWF1 E-box4-e (MT)	Forward	TTTTTAAAAATGAAAAAAAAGCAGG	
	Reverse	TTTTCCTGCTTTTTTTTCATTTTT	
DWF1 E-box5/6-e (MT)	Forward	TTTTTTATATAAAAAAAAAAATTTAT	
	Reverse	TTTTTATAAATTTTTTTTTTTATAT	
UBQ5-q (AT3G62250)	Forward	AAGGCGAAGATCCAAGACAA	qPCR
	Reverse	ACTCCTTCCTCAAACGCTGA	
DWF1-q (AT3G19820),	Forward	TCGCAAGGAACTCTTGGACT	
	Reverse	AACCACCATCCCACATTGTT	
DWF4-q (AT3G50660),	Forward	CGGAGCAAATTCTCGATCTC	
	Reverse	TCTTTGAGTGCTTTGCGATG	
CPD-q (AT5G05690)	Forward	ATGAAAAGGAGGGAGGAGGA	
	Reverse	GAGAAATTTGACGGCGAGAG	
CYP85A2-q (AT3G30180)	Forward	AAGCCCAACCATGATGAAAG	
	Reverse	GGACCGATAGAGTTCCCACA	
DWF1-semi-q (AT3G19820)	Forward	ATGTCGGATCTTCAGACACCGCTTG	Semi qRT-PCR
